# Straightforward synthesis of gold nanoparticles by adding water to an engineered small dendrimer

**DOI:** 10.3762/bjnano.11.95

**Published:** 2020-07-28

**Authors:** Sébastien Gottis, Régis Laurent, Vincent Collière, Anne-Marie Caminade

**Affiliations:** 1 Laboratoire de Chimie de Coordination du CNRS, 205 Route de Narbonne, BP 44099, 31077 Toulouse Cedex 4, France; 2 LCC-CNRS, Université de Toulouse, CNRS, Toulouse, France; 3 Laboratoire de Réactivité et Chimie des Solides, UMR CNRS/UPJV 7314, 33 rue St Leu, 80039 Amiens cedex 1, France

**Keywords:** colloidal suspension, complexation, dendrimer, gold nanoparticle, phosphorus

## Abstract

A small water-soluble phosphorus-containing dendrimer was engineered for the complexation of gold(I) and for its reduction under mild conditions. Gold nanoparticles were obtained as colloidal suspensions simply and only when the powdered form of this dendrimer was dissolved in water, as shown by transmission electron microscopy (TEM) and energy dispersive X-ray spectroscopy (EDX) analyses. The dendrimers acted simultaneously as mild reducers and as nanoreactors, favoring the self-assembly of gold atoms and promoting the growth and stabilization of isolated gold nanoparticles. Thus, an unprecedented method for the synthesis of colloidal suspensions of water-soluble gold nanoparticles was proposed in this work.

## Introduction

Research on nanoparticles (NPs) in general, and gold nanoparticles in particular, results in the generation of thousands of publications every year, including reviews [1–2]. Different applications for gold nanoparticles have been proposed, for example, in catalysis [3–4] and in biology [5] – especially for bio-imaging and cancer therapy [6–7]. In most cases, the synthesis of gold nanoparticles is carried out by the reaction between HAuCl_4_ and a reducing agent (in particular NaBH_4_) in the presence of a suitable compound to simultaneously prevent the aggregation of the nanoparticles and to stabilize them [8–9]. AuCl(tht) (tht = tetrahydrothiophene) has also been shown to be an interesting precursor of gold nanoparticles, but only in a few cases [10].

Among the stabilizing agents, dendrimers [11–17] have long emerged as a powerful stabilizer not only for nanoparticles in general [18–19] but also specifically for gold nanoparticles [20–22]. Indeed, due to the well-defined three-dimensional structure of dendrimers they are suitable templates for the synthesis of nanoparticles in the presence of a reducing agent [23] and prevent nanoparticle aggregation and oxidation [24]. Different types of dendrimers have been used for the stabilization of nanoparticles, in particular poly(amidoamine) (PAMAM) [25] and poly(propylene imine) (PPI) [26]. Phosphorus-containing dendrimers [27–29] have also been used to stabilize different nanoparticles made from palladium [30], platinum [31], ruthenium (in the presence of a reducer) [32], titanium oxo-clusters [33–34] and even from crystals of Au_55_ gold clusters [35–36]. In most cases, the oxidation state of the metal precursor was either zero (Pd^0^, Pt^0^, Au^0^) or four (Ti^IV^O_2_ clusters) and no change in the oxidation state happened in the nanoparticle metal precursor, except in the case when a reducer was used for the Ru nanoparticle synthesis.

Commercially available gold nanoparticles are sold as colloidal suspensions that are generally dissolved either in water or in buffer. As an alternative to this commonly used method, it would be convenient to have a stable solid precursor, which could almost instantly produce gold NPs on demand when dissolved in water. The suitable precursor should ideally be a single component with the following prerequisites: i) be able to complex gold(I); ii) be a mild reductant; iii) induce the solubility in water; and iv) stabilize the nanoparticles once they are formed. In this context, specifically designed dendrimers could meet all these requirements.

Among all the dendrimers that have been synthesized, those incorporating P=N–P=S linkages are of particular interest in the field of nanoparticle synthesis due to the reactivity of this linkage [37–39] and due to its ability to complex metals, especially gold(I), with the sulfur atom [40–41]. A recent theoretical work demonstrated that the highest occupied molecular orbital of a small dendrimer containing the P=N–P=S linkage is located in this linkage with a noticeable electronic delocalization [42]. Thus, the presence of P=N–P=S linkages in the precursor is desirable. The precursor should induce the persistent colloidal stability of the nanoparticles in water. It has been already shown that the Girard’s T reagent (acethydrazide trimethylammonium chloride), used as a terminal function in dendrimers, can induce dendrimer solubility in water, allowing the colloidal stability of nanolatex covered by such a function and also the formation of structured hydrogels [43–44]. Hydrazine is a well-known reductant, which has been used for the seeded growth of gold NPs [45]. Some derivatives of hydrazine, such as the phenylhydrazine, are also used as a reductant [46]; however, to the best of our knowledge, there is no report on the use of Girard’s reagents as a reductant. It is known that when in water the hydrazones and acylhydrazones are in equilibrium with their hydrolyzed forms (aldehyde and hydrazine), a property which is particularly useful for the building of combinatorial libraries [47–48]. However, this reaction can be largely shifted toward the hydrazone form. The Girard’s reagent linked to the dendrimer might act as a water-solubilizing function, as structuring agent in water media, and eventually as a reductant if released (at least in part) when in solution. In addition, it is important to mention that the compound should be obtained after a minimum number of steps. Thus, it should be small while having a branched structure for a better stabilization of the gold nanoparticles, as it will be shown in this paper.

## Results

### Synthesis and characterization of dendrimers

**Figure 1:**
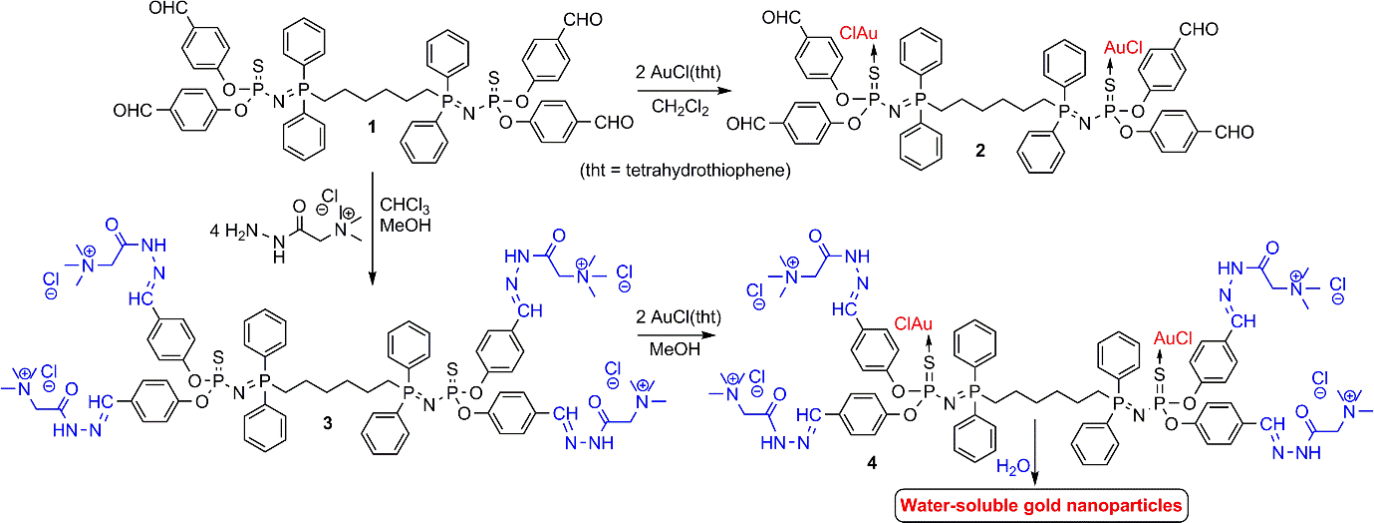
Synthesis of the small phosphorus dendrimers and their complexation with gold toward the synthesis and stabilization of gold nanoparticles in water.

The potential reducing ability of the Girard’s T reagent toward AuCl(tht) was verified in water prior to the synthesis and functionalization of the dendrimers. A reaction was rapidly observed at room temperature showing that this reagent is indeed able to reduce gold. However, the final solution was black and, therefore, there is no nanoparticle, as shown by UV–vis spectroscopy. Thus, the results suggest that the Girard’s T reagent is a stronger reductant than the amino acids such as ʟ-histidine, which have been used to synthesize gold nanoparticles at a higher temperature (80 °C) [49], or cysteine [50]. The compound **1** was the dendrimer chosen for the functionalization with the Girard’s T reagent. The synthesis was performed via the Staudinger reaction between 1,6-bis(diphenylphosphino)hexane and a phosphorus azide functionalized by two aldehydes, as previously published [37]. The ability of compound **1** P=N–P=S linkages to complex gold and to react with AuCl(tht), yielding compound **2**, was demonstrated by a broadening and dramatic signal shift corresponding to the P=S group in the ^31^P nuclear magnetic resonance (NMR) spectra (Figure 1 and Table 1). Indeed, this signal shifted from 52.1 ppm in **1** to 33.7 ppm in **2** (Δδ = −18.4 ppm). The attempts to generate the gold nanoparticles from compound **2** were carried out by adding water. This compound was not very soluble in water and no change in the solution color was observed. Therefore, it was concluded that no reaction occurred and the gold nanoparticles were not generated.

**Table 1:** Comparison of the ^31^P {^1^H} NMR data for all the generated compounds.

Compounds	δ P=N (ppm)	δ P=S (ppm)	^2^*J*_PP_ (Hz)

**1**^a^	20.8	52.1	35
**2**^a^	23.2	33.7	27
**3**^b^	21.3	50.6	29
**4**^c^	23.8	33.2 (br)	20

^a^in CDCl_3_; ^b^in D_2_O; ^c^in MeOH (with a capillary of C_6_D_6_).

The second step in the precursor synthesis was the condensation reaction between the aldehydes of compound **1** with the Girard’s T reagent, yielding compound **3** as a white powder. The ^31^P NMR spectra of compound **3** were different depending on the solvent used. When D_2_O was used as the solvent, only the expected set of doublets was observed (21.3 (P=N) ppm and 50.6 (P=S) ppm, *J*_PP_ = 29 Hz, Figure S5, Supporting Information File 1). However, when DMSO was the solvent used, several sets of doublets were observed within the same region (Supporting Information File 1, Figure S6) presumably due to the presence of both the *Z* and *E-*isomers of the hydrazones [51]. These isomers were also detected in the ^1^H NMR spectra as two signals corresponding to the CH=N groups (8.13 ppm and 8.42 ppm) when DMSO was used as the solvent. The reaction completion was confirmed by the disappearance of the signal corresponding to the aldehydes in the ^1^H and ^13^C NMR spectra. The presence of isomers resulting from the CH=N–NHC(O)CH_2_NMe_3_ linkage was confirmed in the ^13^C NMR spectra by the presence of two signals for each carbon atom present in this linkage. The complexation of the dendritic compound **3** with AuCl(tht), yielding compound **4** as a white powder, was carried out following the same procedure used to obtain compound **2** from compound **1**. Compound **4** was characterized in the ^31^P NMR spectra as a broad signal at 33.2 ppm (P=S–Au, Δδ = −17.4 ppm) and a doublet at 23.8 ppm (P=N, Δδ = +3 ppm) (Table 1). Modifications of the ^31^P NMR chemical shifts are frequently connected with modifications in the (crystallographic) cone angles. It has been shown, in particular, that a decrease in the cone angle of the phosphine complexes induces a shielding of the ^31^P NMR signal [52]. A decrease in the cone angle of the substituents around the P=S bond in the P=N–P=S linkages [53] upon complexation with gold [54] can be associated with the observed shielding of the ^31^P NMR chemical signal. In addition, it has been shown that the ^2^*J*_PP_ coupling constant values decrease when the electron-withdrawing power of the substituents decreases [55]. The density functional theory (DFT) calculations on free and Au-complexed P=N–P=S linkages have shown that a charge is transferred from the gold atom to the sulfur atom and thus the electron-withdrawing power of the sulfur towards phosphorus decreases [42]. This is consistent with the decrease observed for the ^2^*J*_PP_ coupling constant upon complexation (Table 1).

### Synthesis of gold nanoparticles and UV–vis characterization

**Figure 2:**
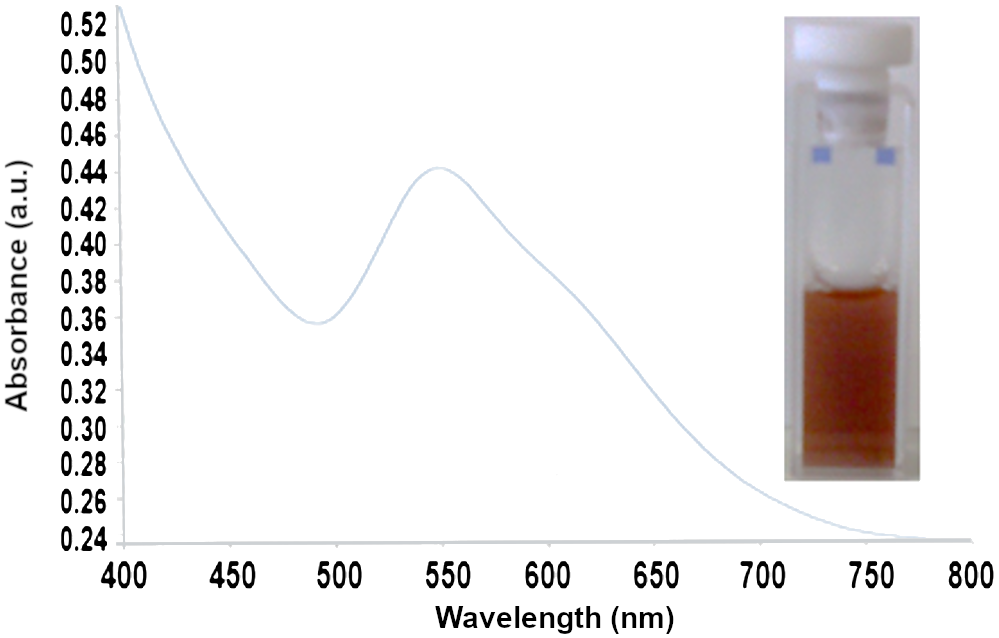
The visible spectrum of the colloidal suspension of Au NPs in water (inset on the right) obtained from compound **4**.

The dissolution of compound **4** in organic solvents such as methanol or DMSO yielded colorless solutions. On the other hand, when the white powdered form of compound **4** was dissolved in water it instantaneously led to a deep red colloidal suspension, as shown in Figure 2. The red color was a strong indicator of the presence of gold nanoparticles since this color corresponds to the surface plasmon resonance wavelength. This is a well-known phenomenon observed in gold nanoparticles [56]. Considering that the gold nanoparticles are spherical, the maximum intensity of the visible spectrum at 545 nm should correspond to a mean size of ≈50 nm for the gold nanoparticles [57–58]. The shoulder detected at ≈630 nm corresponds to the longitudinal surface plasmon resonance, and it is characteristic of the presence of non-spherical gold NPs, in particular rod-like or triangular NPs [59–60]. The presence of this shoulder shifted the absorption maximum toward the red wavelength region, suggesting that the approximate diameter of the gold nanoparticles should be smaller than 50 nm.

### Characterization of gold nanoparticles by transmission electron microscopy

**Figure 3:**
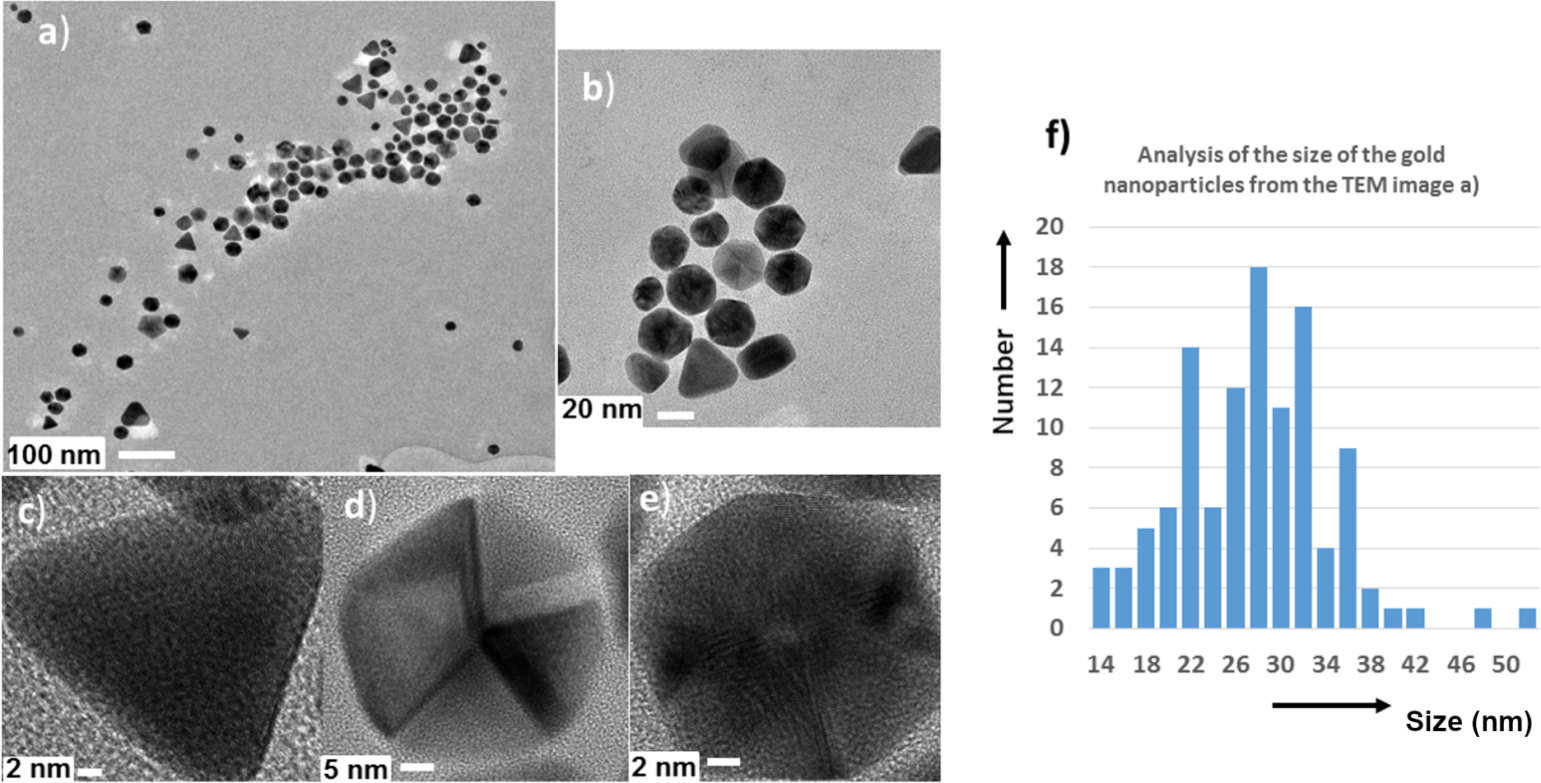
(a–e) TEM images of the gold nanoparticles obtained from the dendrimer compound **4** at different magnifications. (f) Analysis of the size distribution of the gold NPs from the TEM image (a). Scale bars: 100 nm (a), 20 nm (b), 2 nm (c and e) and 5 nm (d).

To confirm the spontaneous formation of the gold NPs, transmission electron microscopy (TEM) images were obtained from a drop of a colloidal suspension of compound **4** in water (Figure 3). Figure 3A displays images of the NPs with diameters ranging from 20 to 50 nm (mean value is 28 nm, Figure 3F). It can be seen from Figure 3B that these nanoparticles have a variety of slightly different shapes. Figure 3C provides a detailed view of a triangular nanoparticle in which the layers of the gold atoms in the nanocrystals can be seen. Figure 3D displays a pentagonal nanoparticle which is constituted by five associated triangles. Figure 3E shows a hexagonal nanoparticle with a shape similar to a spherical desert rose crystal also constituted by associated triangles. An enlargement of Figure 3C is given in Figure S1 (Supporting Information File 1), from which an Au–Au distance of ≈2.96 Å can be measured. This distance is shorter than what has been seen for the gold NPs and fibers stabilized by amines (Au^I^, ca. 3.25 Å) [10], but slightly longer than in the case of gold particles in silica glass and gold foil (2.84 Å) [61] or in bulk gold (2.88427 Å) [10].

The dependence between the gold nanoparticle shape and the conditions in which they were generated is the subject of a few studies [62–64]. According to these studies none of the nanoparticles were obtained as a resulted of fast nucleation, which in turn should lead to spherical and uniform nanoparticles with a 2–5 nm diameter range. On the contrary, the shapes that were observed here were obtained through a controlled two-step process named “seed-mediated growth”. The first step of this process is the generation of very small spherical nanoparticles that serve as seeds when the conditions are modified by adding more gold and another reductant [64]. For this work a single component was used to obtain the shape-controlled nanoparticles (triangles and associated triangles) in an unprecedented way.

### Characterization of gold nanoparticles by energy-dispersive X-ray spectroscopy

**Figure 4:**
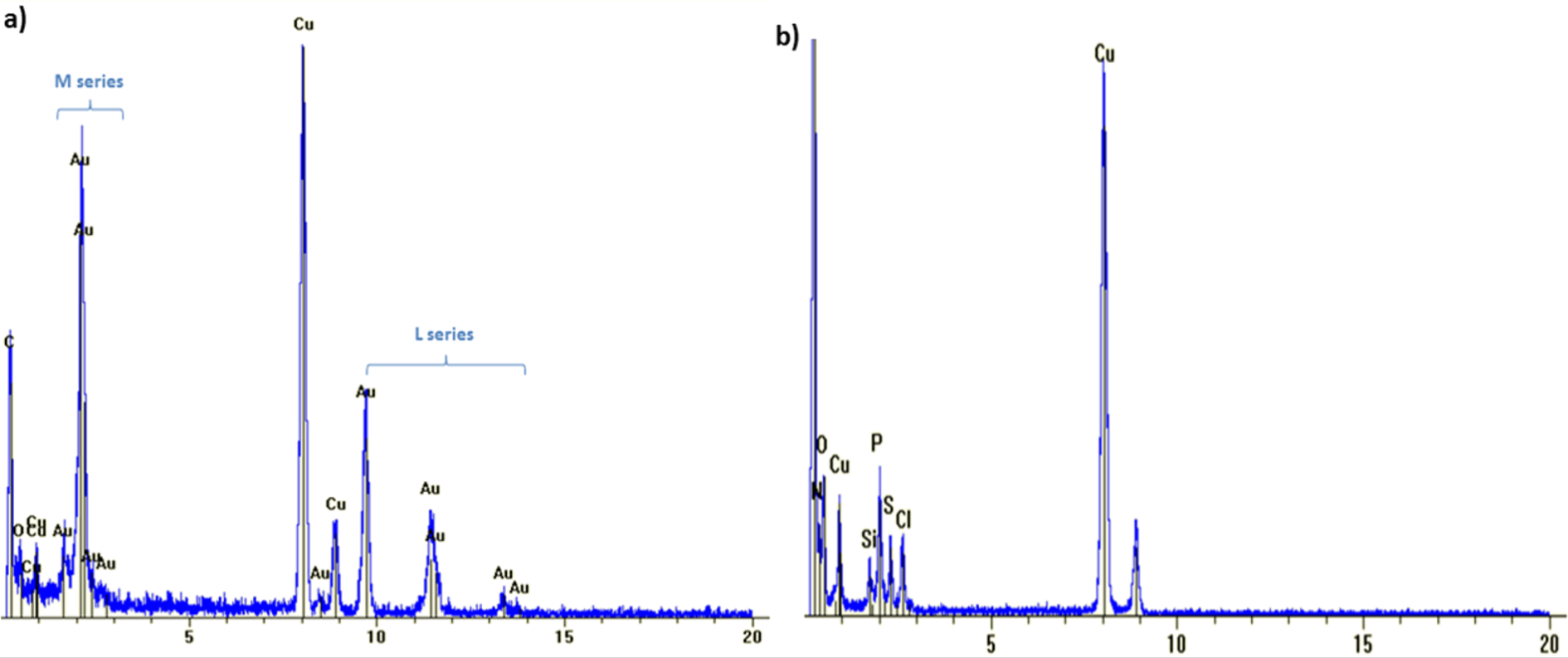
EDX spectra (a) for the gold nanoparticles and (b) for the background. The copper comes from the grid used during the experiments.

In order to determine the presence and location of the different elements energy-dispersive X-ray spectroscopy (EDX) analysis was also performed in parallel with TEM. All the characteristic X-ray lines expected from the gold L and M series were observed when focusing on the gold nanoparticles (Figure 4A). The signals obtained from the background (close to the nanoparticles) showed phosphorus and sulfur atoms both coming from the dendrimers but no gold was detected (Figure 4B). This result shows that the gold from compound **4** has been entirely used for the generation of the gold NPs which were stabilized at their surface by the dendrimers, through their P=S groups.

## Discussion

The first step that dictates the process of generating the gold nanoparticles is the equilibrium of hydrazone/hydrazine. Indeed, the generation of the gold NPs from compound **4** occurs only in water which is known to slowly hydrolyze hydrazones. It is important to highlight that no nanoparticle was generated in water from compound **2** which does not contain hydrazone linkages. The hydrazone/hydrazine equilibrium was also seen when compound **3** was placed into DMSO-containing water, leading to the reappearance of a small signal corresponding to the aldehydes in the ^1^H NMR spectrum (Figure S9, Supporting Information File 1). The released Girard's T reagent should act as a reducing agent, as was shown in a preliminary experiment. In this work, in the case in which compound **4** was used, only a small amount the of Girard’s T reagent was present in the colloidal suspension. The reduction mechanism of Au(I) to Au(0) leading to the gold nanoparticles was not well understood until a theoretical work was published in 2015 [65]. In this work, it was demonstrated that the first step required for the nonradical formation of an Au–Au bond was the presence of hydrogen followed by the insertion of a third gold atom to form a triangle by self-assembly. The release of only a small quantity of the Girard’s T reagent was sufficient to induce the first nucleation steps. The cleavage of all the hydrazone linkages would generate dendrimers **1** or **2** that are insoluble in water; however, no precipitate was observed. The intact dendrimer **4**, on the other hand, acted as a nanoreactor in water favoring the gold atoms to self-assemble, promoting growth and stabilization of the NPs in particular through the sulfur atoms present on the dendrimers. The dendrimer also ensures the solubility of the nanoparticles in water, forming colloidal suspensions. Each dendrimer can act as a bridge at the surface of the gold NPs, increasing their stabilization due to the presence of two P=S groups. The absence of gold outside the nanoparticles, according to the EDX results for the background, indicated that the gold nanoparticles were obtained in a quantitative yield. The conditions for clustering the gold NPs were recently studied [66] and it was shown that heating the gold NPs at a very high temperature (800 °C) yielded NPs with a pentagonal symmetry [67]. In this work a very similar structure was obtained, as shown in Figure 3D; however, due to the presence of the dendrimers the NPs were obtained here at room temperature. Even though a better control of the polydispersity is desirable, other gold nanoparticles of relatively high dispersity have been recently used for drug delivery in three different cell lines [68].

## Conclusion

In conclusion, we have shown for the first time that a single compound based on an engineered small dendrimer was able to produce shape-controlled gold nanoparticles (triangles and other triangle-associated shapes), simply by adding water, at room temperature. This precursor (dendrimer **4**) is a very stable white powder that can be safely stored for months (even years) at room temperature on a shelf in a closed flask and does not require any specific reagents to generate the gold nanoparticles, such as a reducing agent, heating or the presence of gold.

This is a novel concept as it only requires the addition of water for the synthesis of the nanoparticles. This is a safer and an environmentally viable alternative to all the previous work related to the synthesis of gold nanoparticles in aqueous colloidal suspensions. The synthesis process shown here is based on the use of an original small dendrimer which acts simultaneously as a mild reducing agent and as a nanoreactor for the self-assembly of gold atoms, stabilizing the gold nanoparticles and inducing their water solubility forming colloidal suspensions. It must be emphasized that currently there is no other example of a comparable process in the literature.

Possible extensions of this work should consider using larger (but more expensive) dendrimers and polymers to synthesize NPs with different shapes/sizes and degrees of polydispersity. In addition, other metals should also be considered as alternatives to synthesize NPs.

## Experimental

### General synthesis and characterization procedures

**Figure 5:**
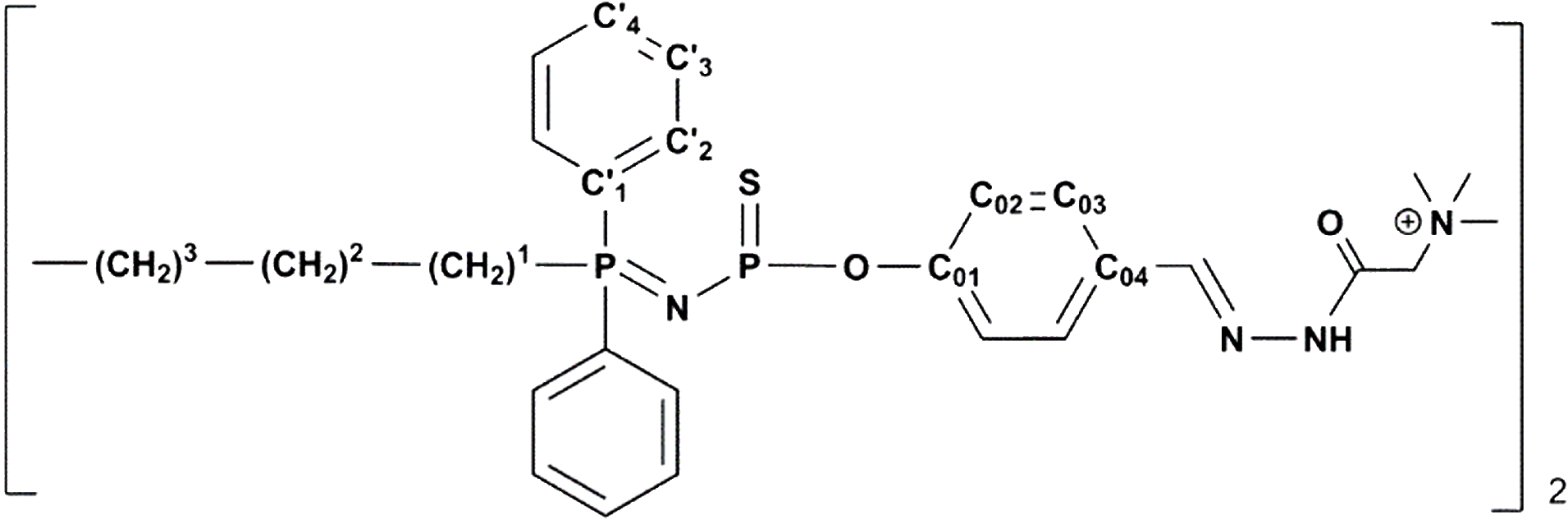
Numbering used for the NMR assignment.

Girard’s T reagent was used as purchased from Sigma-Aldrich (St. Louis, MO, USA). AuCl(tht) was synthesized according to a published procedure [69]. Compound **1** was synthesized as previously reported [37]. All reactions were carried out under an argon atmosphere using the standard Schlenk techniques. All the solvents were distilled (pentane over phosphorus pentoxide, and CH_2_Cl_2_ over CaH_2_). ^1^H, ^13^C, and ^31^P NMR spectra were recorded with Bruker ARX 250, AV300 or DPX 300 spectrometers (Wissembourg, France). The references for the NMR chemical shifts are 85% H_3_PO_4_ for ^31^P NMR, SiMe_4_ for ^1^H and ^13^C NMR. The numbering used for the NMR assignments is depicted in Figure 5.

### Synthesis and characterization of dendrimer complex **2**

107 mg (9.79 × 10^−2^ mmol) of **1** and 73.31 mg (0.229 mmol; 2.3 equiv) of AuCl(tht) (in dry CH_2_Cl_2_) were solubilized in a 10 mL round-bottom flask under argon atmosphere at room temperature. The solution was stirred for 1 h and evaporated until dry. Then the product was rinsed with CH_2_Cl_2_/pentane (1:5) and filtered. Next, the crude product was evaporated until dryness in order to obtain 152.5 mg (quantitative yield) of the complex **2** as a white foam. ^31^P {^1^H} NMR (101 MHz, CDCl_3_) 23.2 (d, ^2^*J*_PP_ = 27 Hz, P=N), 33.7 (d, ^2^*J*_PP_ = 27 Hz, P=S); ^1^H NMR (250 MHz, CDCl_3_) 1.44 (br s, 8H, CH_2_), 2.83 (m, 4H, (CH_2_)^1^), 7.33 (d, ^3^*J*_HH_ = 8 Hz, 8H, C_02_H), 7.53 (m, 8H, C’_2_H), 7.63 (m, 12H, C’_3_H, C’_4_H), 7.87 (d, ^3^*J*_HH_ = 8 Hz, 8H, C_03_H), 9.98 (s, 4H, CHO); ^13^C {^1^H} NMR (75 MHz, CDCl_3_) 21.4 (d, ^3^*J*_CP_ = 3.7 Hz, (CH_2_)^3^), 26.9 (d, ^1^*J*_CP_ = 65.1 Hz, (CH_2_)^1^), 29.8 (d, ^2^*J*_CP_ = 15.9 Hz, (CH_2_)^2^), 122.0 (d, ^3^*J*_CP_ = 5.3 Hz, C_02_), 126.8 (br d, ^1^*J*_CP_ = 110 Hz, C'_1_), 129.3 (d, ^3^*J*_CP_ = 13 Hz, C'_3_), 131.5 (d, ^2^*J*_CP_ = 8 Hz, C'_2_), 131.6 (s, C_03_), 133.4 (s, C'_4_), 133.7 (s, C_04_), 155.1 (br s, C_01_), 190.7 (s, CHO).

### Synthesis and characterization of dendrimer **3**

1.0 g (9.15 mmol) of **1** was solubilized in 50 mL of CHCl_3_ in a 100 mL round-bottom flask. A solution containing 616.5 mg (3.68 mmol; 4.02 equiv) of Girard’s T reagent in 25 mL of MeOH was added dropwise to the aldehyde solution. The mixture was stirred overnight at room temperature under an argon atmosphere. The solvent was then evaporated and the crude product was washed twice with 25 mL of CHCl_3_. 1.548 g (quantitative yield) of **3** were obtained as a white powder. ^31^P {^1^H} NMR (121 MHz, D_2_O) 21.3 (d, ^2^*J*_PP_ = 29 Hz, P=N), 50.6 (d, ^2^*J*_PP_ = 29 Hz, P=S); ^1^H NMR (300 MHz, DMSO-*d*_6_) 1.28 (br m, 8H, CH_2_), 2.80 (m, 4H, (CH_2_)^1^), 3.24–3.38 (m, 36H, CH_3_-N^+^), 4.24 and 4.82 (2s, 8H, CH_2_-N^+^), 7.23 (d, ^3^*J*_HH_ = 8 Hz, 8H, C_02_H), 7.51 (m, 8H, C’_2_H), 7.59–7.75 (m, 20H, C_03_H, C’_3_H, C’_4_H), 8.13 and 8.42 (2s, 4H, CH=N), 12.18 and 13.12 (2s, 4H, NH); ^13^C {^1^H} NMR (75 MHz, DMSO-*d*_6_) 21.4 (br, (CH_2_)^3^), 25.6 (d, ^1^*J*_CP’_ = 65.0 Hz, (CH_2_)^1^), 29.7 (d, ^2^*J*_CP’_ = 16.2 Hz, (CH_2_)^2^), 53.7 and 53.9 (2s, CH_3_-N^+^), 62.7 and 63.8 (2s, CH_2_N), 122.1 (br s, C_02_), 129.3 (m, C'_1_), 129.4 (d, ^3^*J*_CP'_ = 12 Hz, C'_3_), 130.2 (s, C_03_), 131.4 (d, ^2^*J*_CP’_ = 10.9 Hz, C'_2_), 133.0 (br s, C'_4_, C_04_), 145.1 and 148.4 (2s, C=N), 153.7 (m, C_01_), 160.3 and 165.8 (2s, C=O).

### Synthesis and characterization of dendrimer complex **4**

163 mg of **3** and 71.1 mg (2.3 equiv) of AuCl(tht) in 5 mL of MeOH were added to a 10 mL round-bottom flask and solubilized under an argon atmosphere at room temperature. The solution was stirred for 1 h and then evaporated before being rinsed with CH_2_Cl_2_/pentane (1:5) and filtered. Complex **4** was obtained as a white powder (quantitative yield).

^31^P {^1^H} NMR (121 MHz, MeOH/C_6_D_6_) 23.8 (d, ^2^*J*_PP_ = 20 Hz, P=N), 33.2 (d, ^2^*J*_PP_ = 20 Hz, P=S); ^1^H NMR (250 MHz, DMSO-*d*_6_) 1.30 (br m, 8H, CH_2_), 2.81 (m, 4H, (CH_2_)^1^), 3.25–3.46 (m, 36H, CH_3_-N^+^), 4.37 and 4.80 (2s, 8H, CH_2_-N^+^), 7.24 (d, ^3^*J*_HH_ = 8 Hz, 8H, C_02_H), 7.54 (m, 8H, C’_2_H), 7.60–7.75 (m, 20H, C_03_H, C’_3_H, C’_4_H), 8.10 and 8.34 (2s, 4H, CH=N), 12.10 and 12.80 (2s, 4H, NH); ^13^C {^1^H} NMR (75 MHz, DMSO-*d*_6_) 21.4 (br, (CH_2_)^3^), 25.7 (d, ^1^*J*_CP’_ = 65.0 Hz, (CH_2_)^1^), 29.7 (d, ^2^*J*_CP’_ = 16.2 Hz, (CH_2_)^2^), 53.7 and 53.9 (2s, CH_3_-N^+^), 62.7 and 63.7 (2s, CH_2_N), 122.0 (br s, C_02_), 128.9 (m, C'_1_), 129.4 (d, ^3^*J*_CP'_ = 12 Hz, C'_3_), 130.2 (s, C_03_), 131.4 (d, ^2^*J*_CP’_ = 10.9 Hz, C'_2_), 133.0 (br s, C'_4_, C_04_), 145.1 and 148.3 (2s, C=N), 153.7 (m, C_01_), 160.3 and 165.8 (2s, C=O).

### Synthesis and characterization of gold nanoparticles

0.5 mL of water was added to 30 mg of the powdered dendrimer complex **4**. The dissolution occurred promptly yielding a colloidal suspension that instantaneously became deep red. The gold nanoparticles were characterized using a PerkinElmer Lambda 35 UV–vis spectrometer (Waltham, MA, USA). TEM and EDX analyses were carried out on a MET JEOL JEM 2100F – EDS (TEMSCAN, Toulouse) device.

## Supporting Information

The supporting information contains an HRTEM image of a gold nanoparticle showing the gold atomic planes, ^31^P, ^1^H and ^13^C NMR spectra of the compounds **2** and **3**, ^1^H NMR spectra of a slightly hydrolyzed compound **3** and ^31^P, ^1^H and ^13^C NMR spectra of compound **4**.

File 1 Additional HRTEM images and NMR data.
