# Supplementary material for: Straightforward synthesis of gold nanoparticles by adding water to an engineered small dendrimer
Source: Beilstein J Nanotechnol. 2020 Jul 28;11:1110–8. doi: 10.3762/bjnano.11.95 (PMC7404285; doi:10.3762/bjnano.11.95)
Supplement: File 1 — Additional HRTEM images and NMR data. [file Beilstein_J_Nanotechnol-11-1110-s001.pdf]

BEILSTEIN JOURNAL OF NANOTECHNOLOGY

Supporting Information

for

Straightforward synthesis of gold nanoparticles by adding water to an engineered small dendrimer

Sébastien Gottis, Régis Laurent, Vincent Collière and Anne-Marie Caminade

Beilstein J. Nanotechnol. **2020,** *11,* 1110–1118. doi:10.3762/bjnano.11.95

Additional HRTEM images and NMR data

License and Terms: This is a supporting information file under the terms of the Creative Commons Attribution License (http://creativecommons.org/licenses/by/4.0). Please note that the reuse, redistribution and reproduction in particular requires that the authors and source are credited.
The license is subject to the *Beilstein Journal of Nanotechnology* terms and conditions: (https://www.beilstein-journals.org/bjnano)

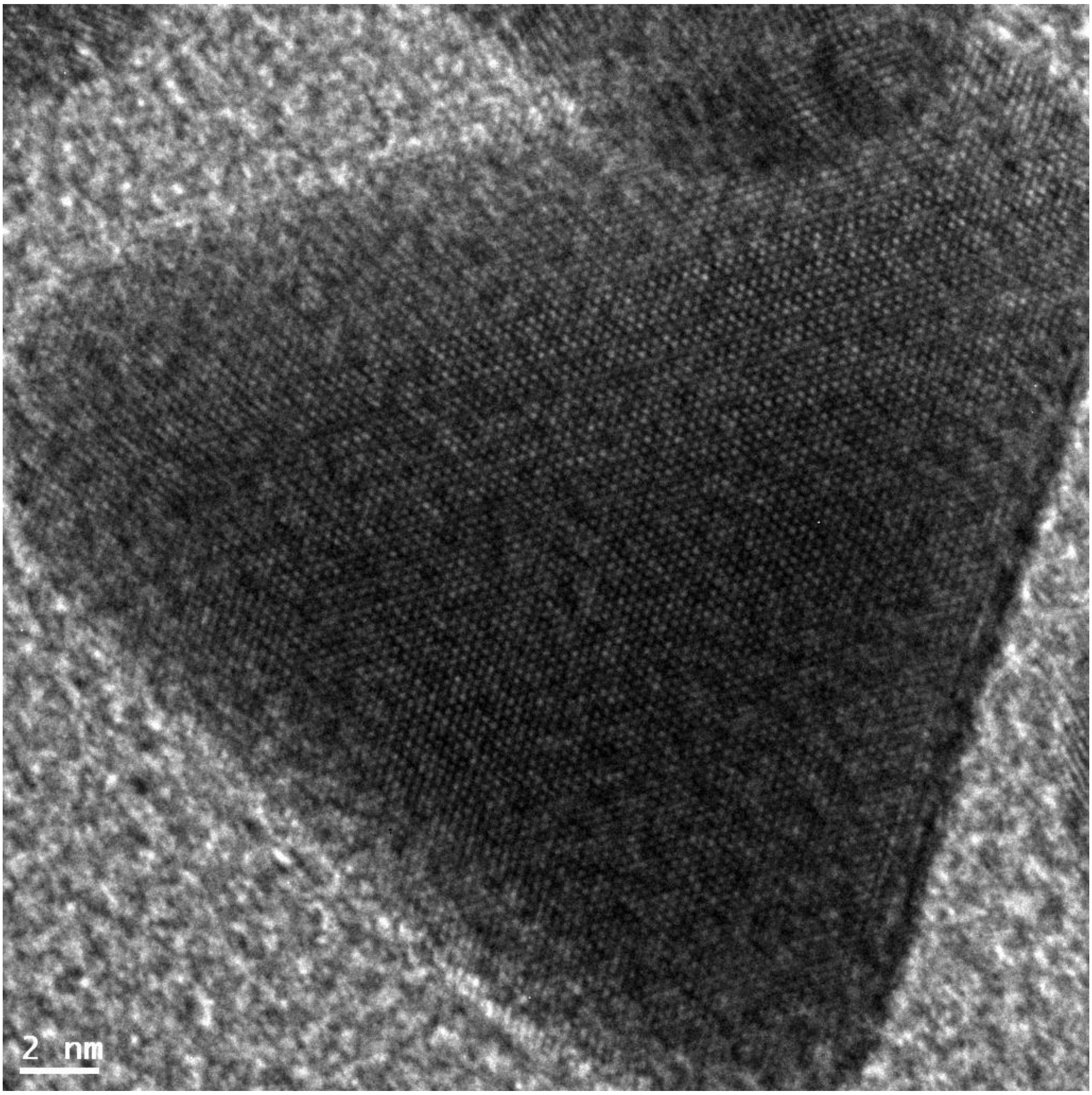

Figure S1: HRTEM image of a gold nanoparticle, showing the atomic planes of gold.

NMR spectra

The ^{31}P, ^{1}H, and ^{13}C NMR spectra of all compounds are given in the following Figures. As usual for dendrimer, all compounds contain traces of solvents, which are detected on the ^{1}H and ^{13}C NMR spectra.

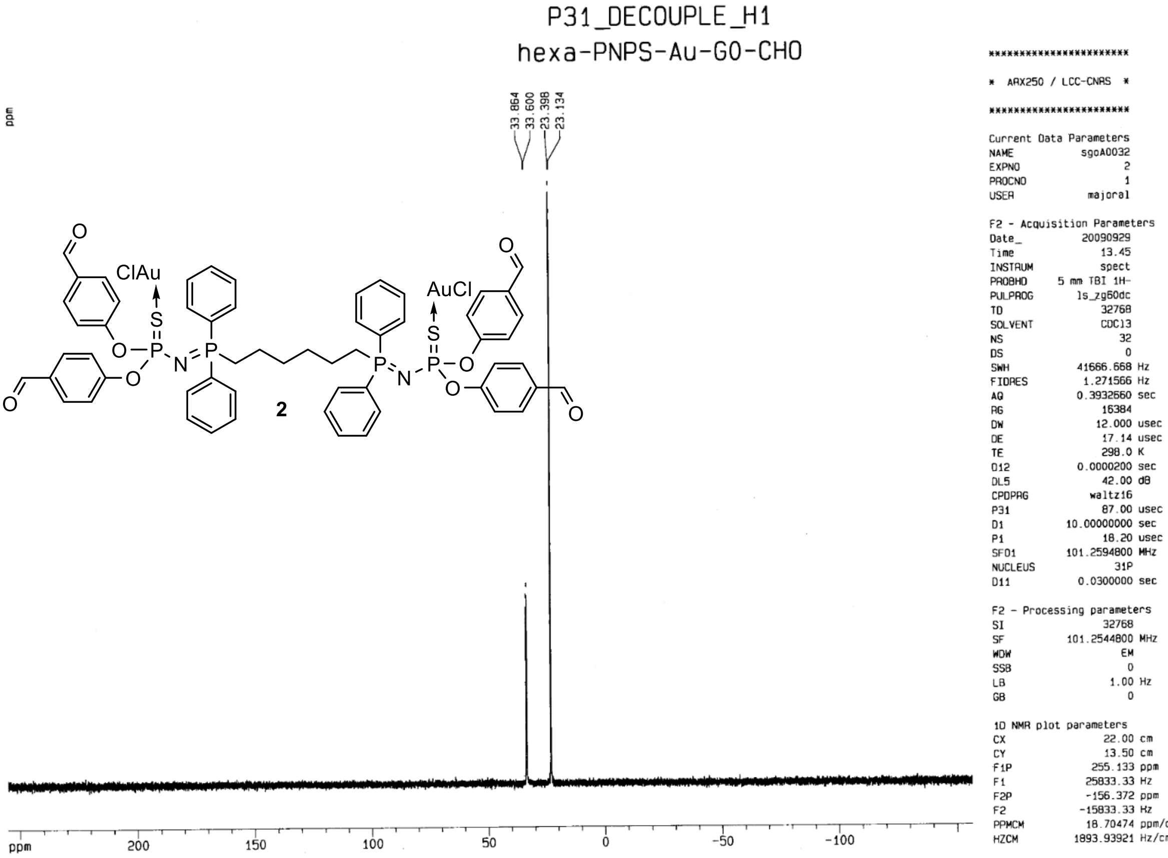

Figure S2: ^{31}P {^{1}H} NMR spectrum of compound **2**.

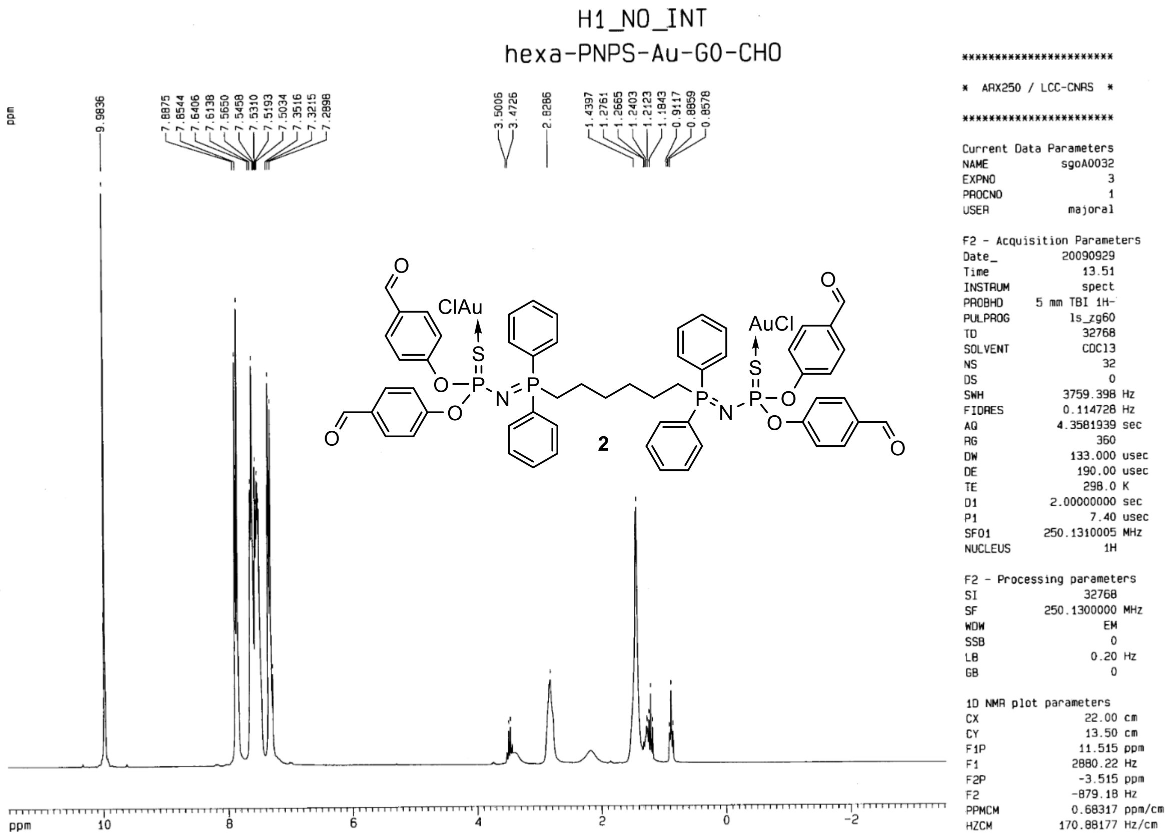

Figure S3: ^{1}H NMR spectrum of compound **2**.

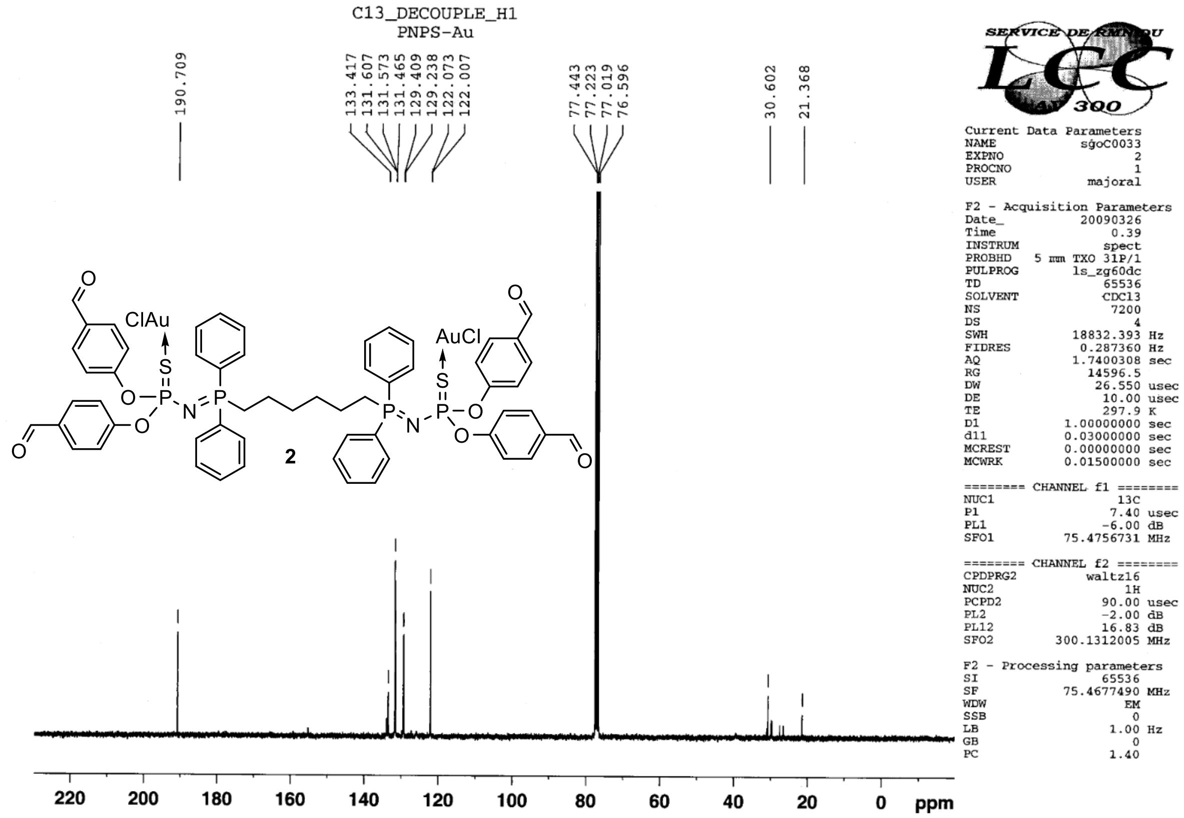

Figure S4: ^{13}C {^{1}H} NMR spectrum of compound **2**.

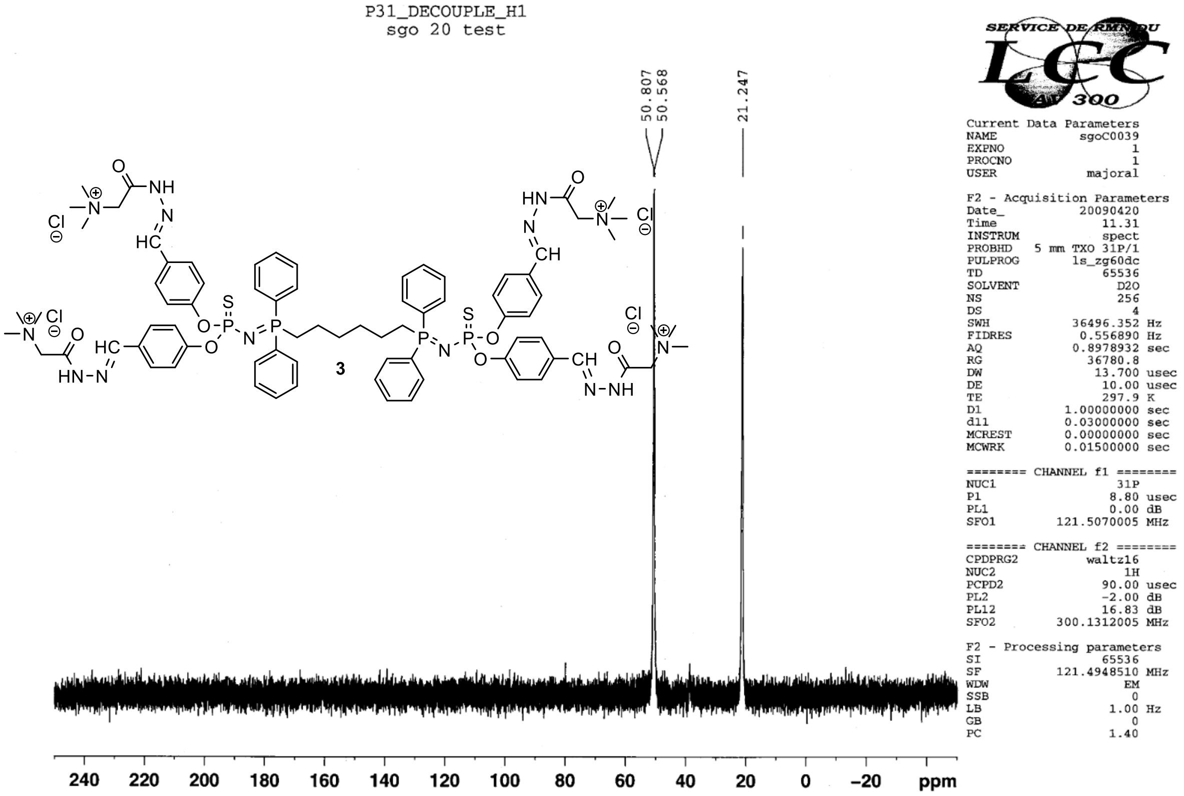

Figure S5: ^{31}P {^{1}H} NMR spectrum of compound **3**.

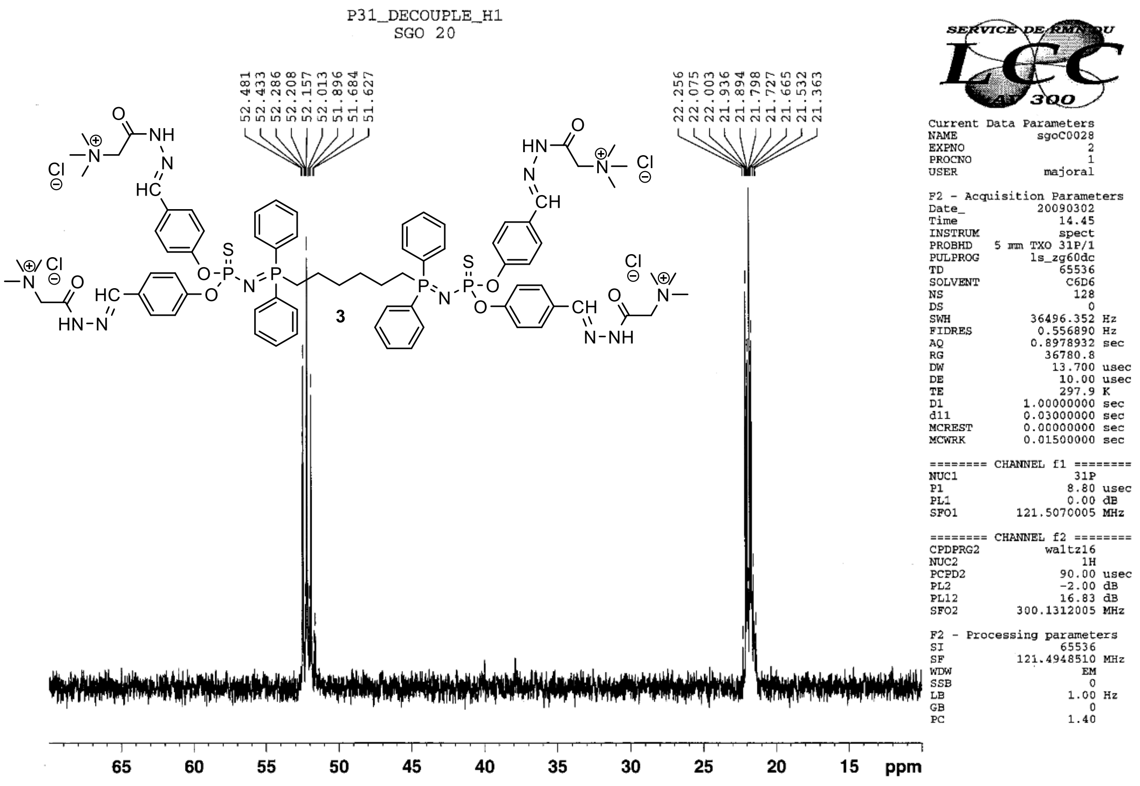

Figure S6: ^{31}P {^{1}H} NMR spectrum of compound **3** in DMSO.

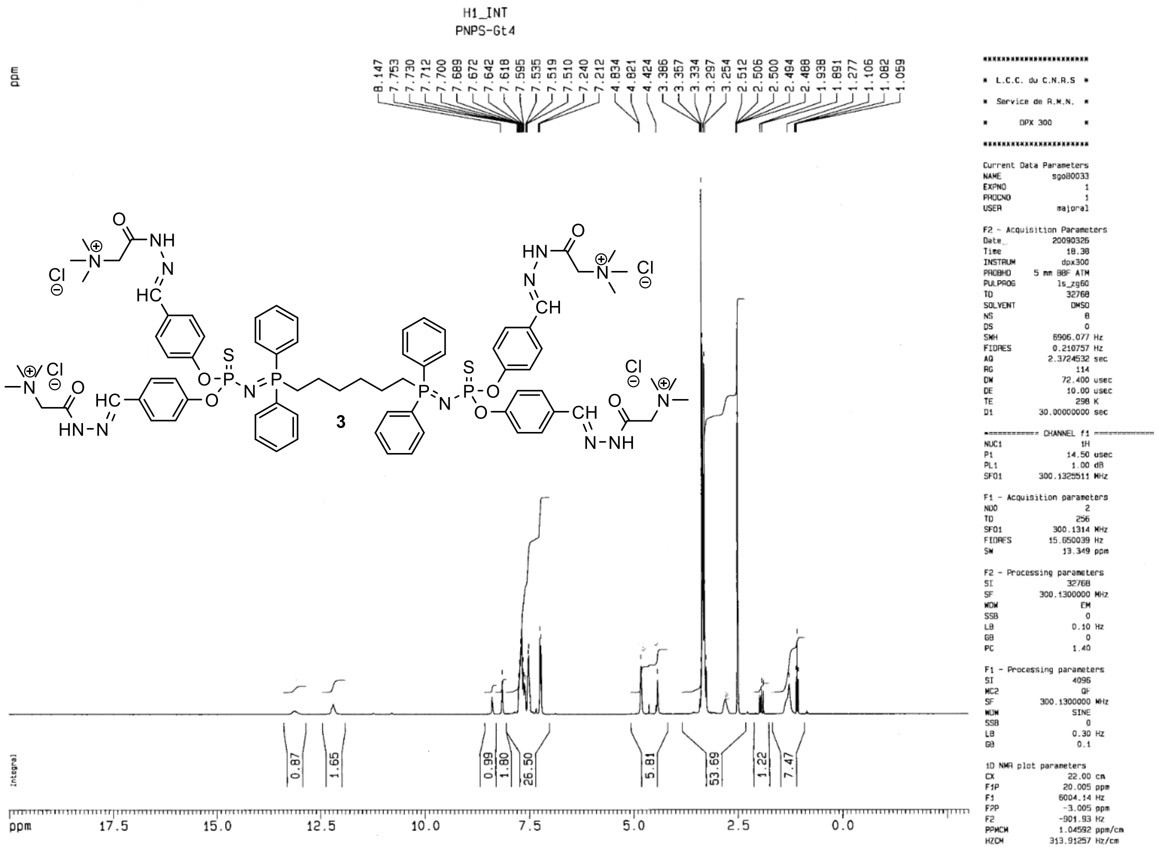

Figure S7: ^{1}H NMR spectrum of compound **3**.

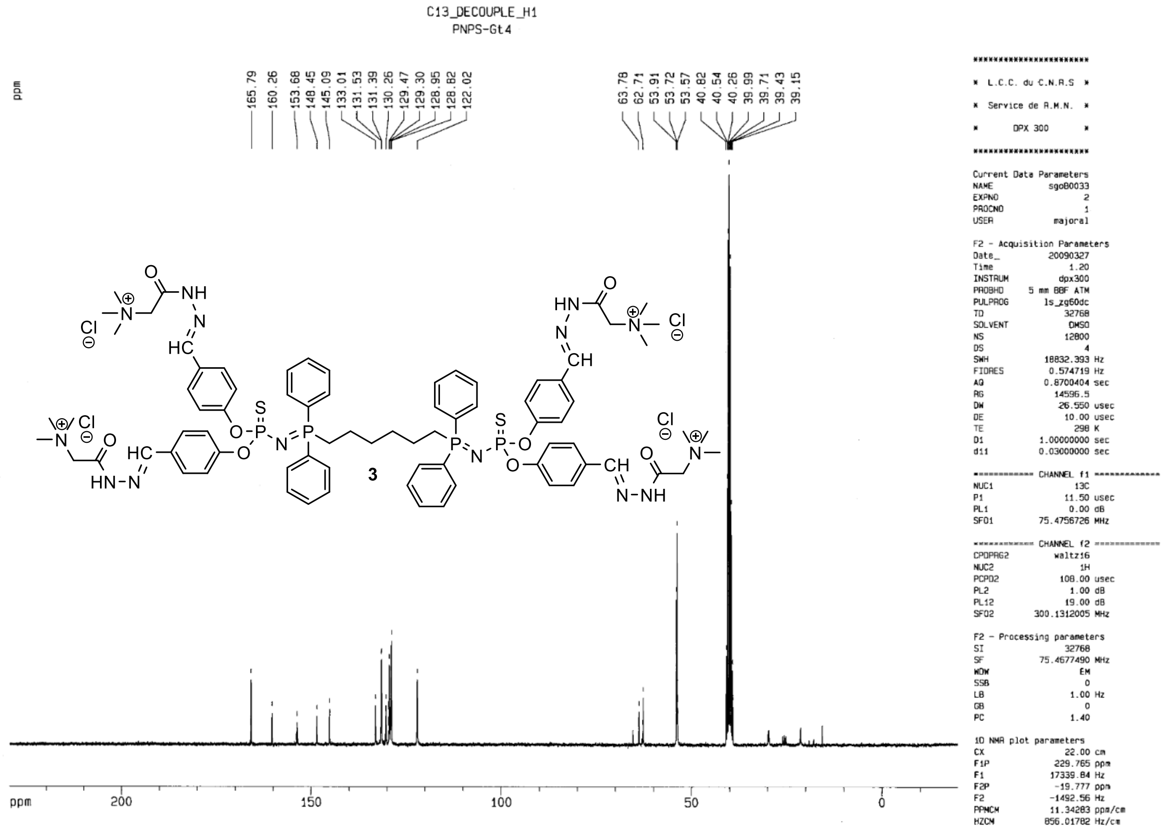

Figure S8: ^{13}C {^{1}H} NMR spectrum of compound **3**.

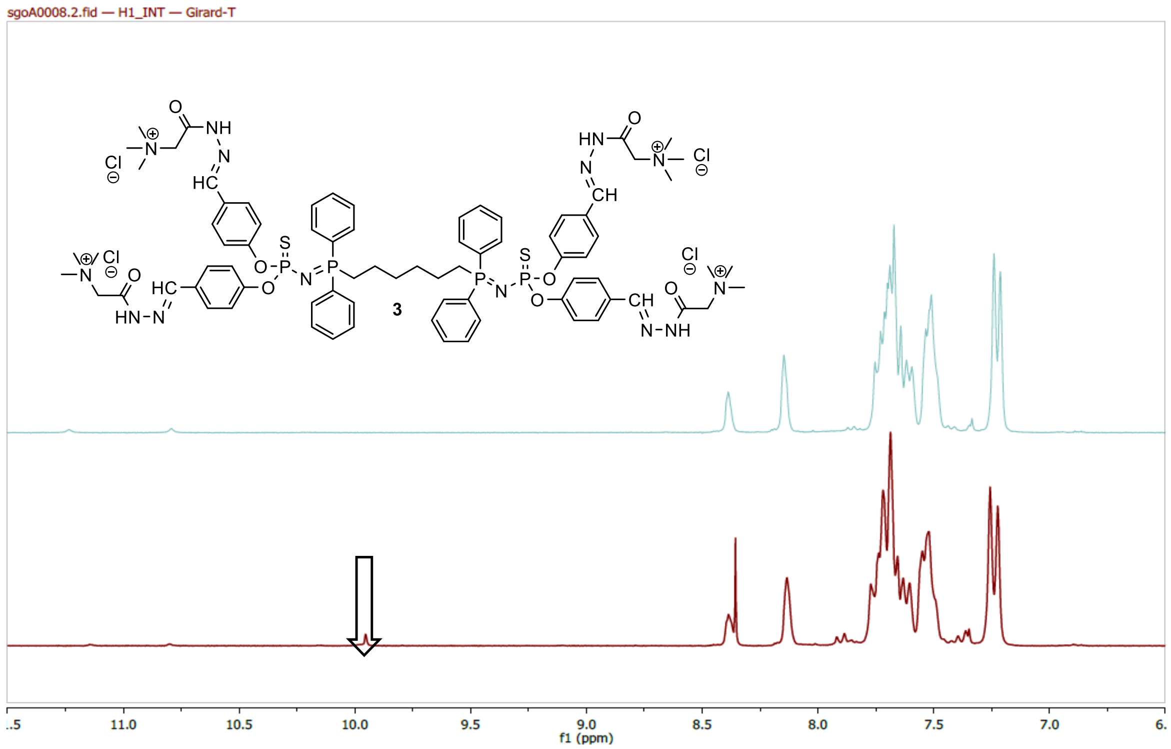

Figure S9: Comparison of an enlargement of ^{1}H NMR spectra of compound **3** pure (green) and slightly hydrolyzed (red) in DMSO and DMSO containing water, showing in particular the re-appearance of the aldehyde signal (arrow).

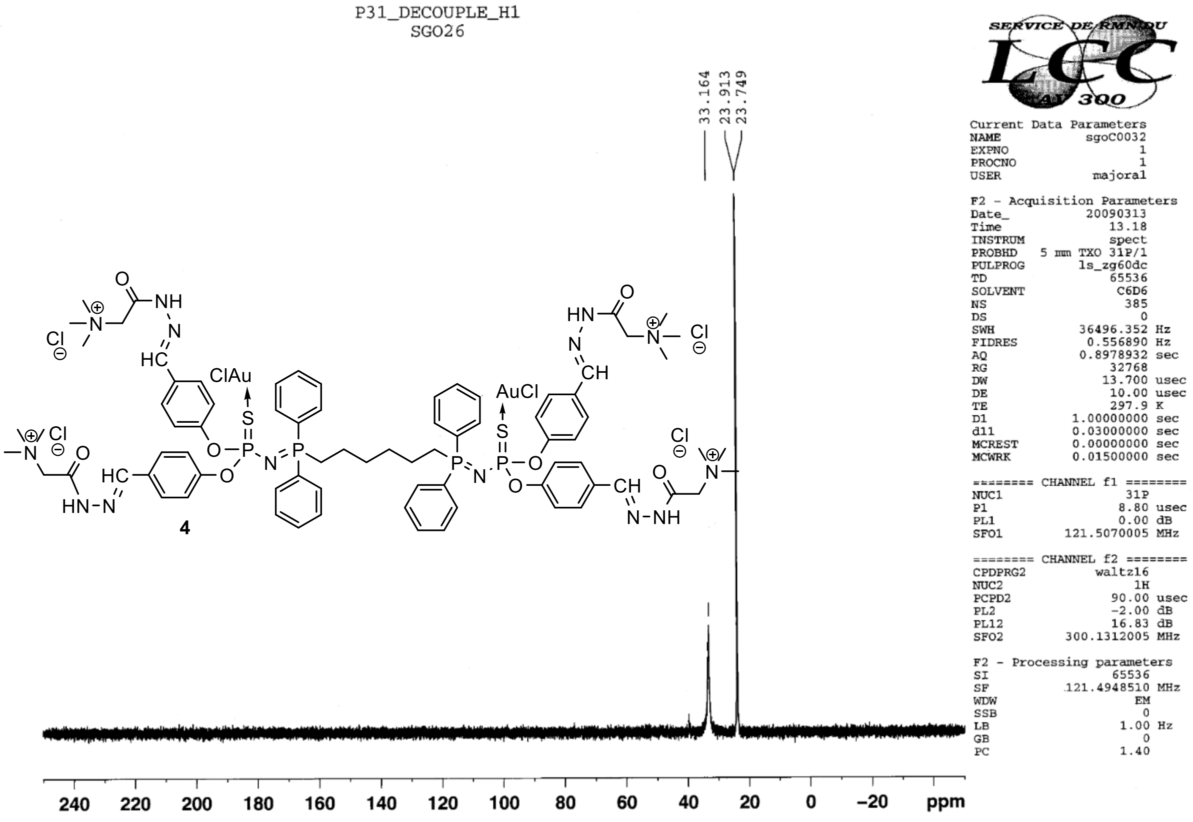

Figure S10: $^{31}P\ \{^{1}H\}$ NMR spectrum of compound **4**.

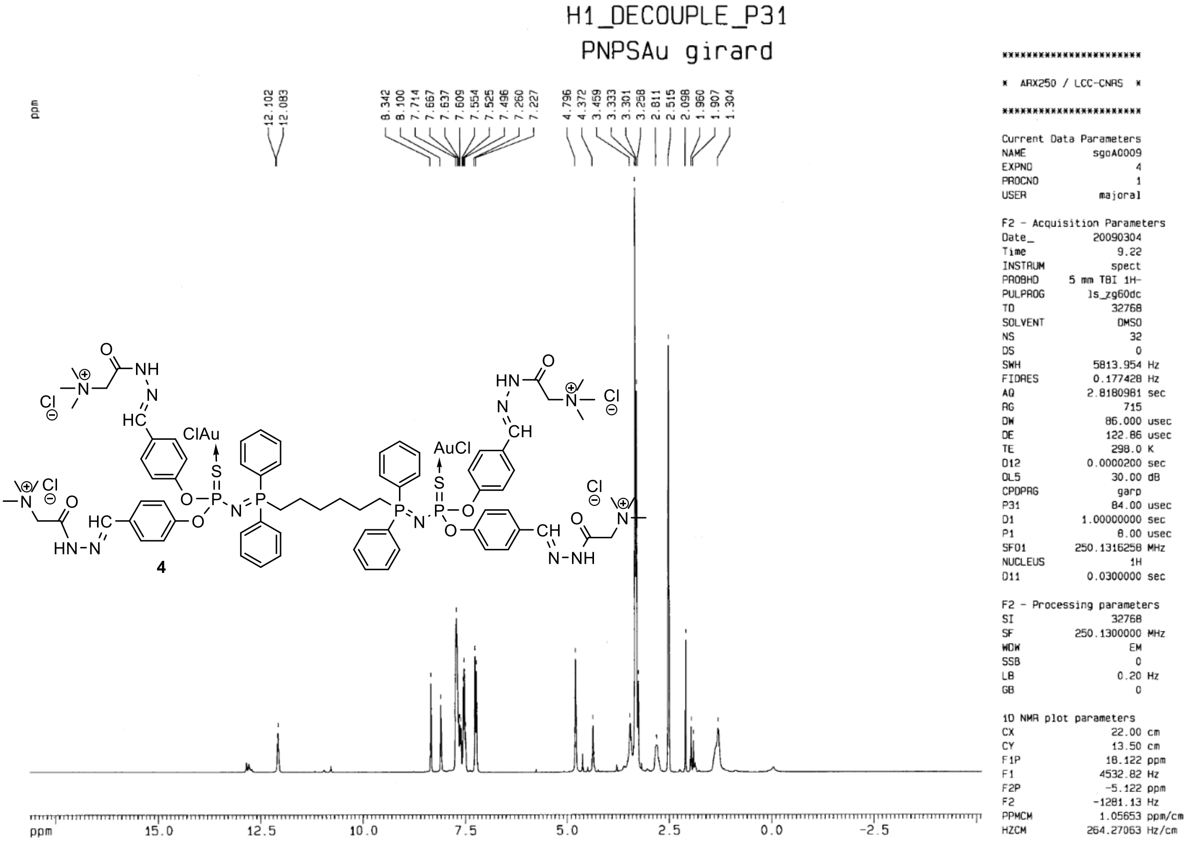

Figure S11: ^{1}H NMR spectrum of compound **4**.

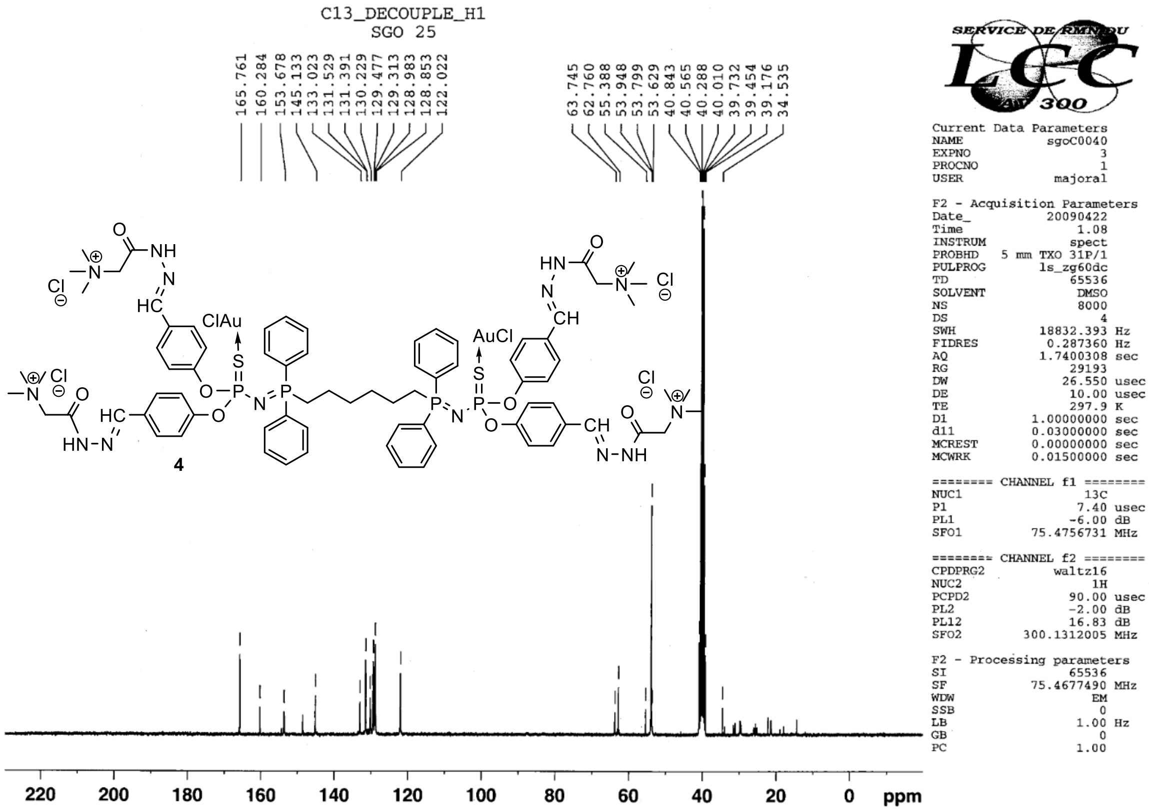

Figure S12: ^{13}C {^{1}H} NMR spectrum of compound **4**.
